# Change in well-being amongst participants in a four-month pedometer-based workplace health program

**DOI:** 10.1186/1471-2458-14-953

**Published:** 2014-09-15

**Authors:** Rosanne LA Freak-Poli, Rory Wolfe, Evelyn Wong, Anna Peeters

**Affiliations:** BakerIDI Heart and Diabetes Institute, Melbourne, Australia; Department of Epidemiology & Preventive Medicine, School of Public Health and Preventive Medicine, Monash University, Melbourne, Australia; Department of Epidemiology, Erasmus MC University Medical Center, Rotterdam, The Netherlands

**Keywords:** Well-being, Happiness, Workplace, Intervention, Evaluation, Physical activity, Prevention

## Abstract

**Background:**

There is increasing uptake of workplace physical activity programs to prevent chronic disease. While they are frequently evaluated for improvement in biomedical risk factors there has been little evaluation of additional benefits for psychosocial health. We aimed to evaluate whether participation in a four-month, team-based, pedometer-based workplace health program known to improve biomedical risk factors is associated with an improvement in well-being, immediately after the program and eight-months after program completion.

**Methods:**

At baseline (2008), 762 adults (aged 40 ± 10 SD years, 42% male) employed in primarily sedentary occupations and voluntarily enrolled in a physical activity program were recruited from ten Australian worksites. Data was collected at baseline, at the completion of the four-month program and eight-months after program completion. The outcome was the WHO-Five Well-being Index (WHO-5), a self-administered five-item scale that can be dichotomised as ‘poor’ (less than 52%) or ‘positive’ (more than or equal to 52%) well-being.

**Results:**

At baseline, 75% of participants had positive well-being (mean: 60 ± 19 SD WHO-5 units). On average, well-being improved immediately after the health program (+3.5 units, p < 0.001) and was sustained eight-months later (+3.4 units from baseline, p < 0.001). In the 25% with poor well-being at baseline, 49.5% moved into the positive well-being category immediately after program completion, sustained eight-months later (p < 0.001).

**Conclusions:**

Clinically relevant immediate and sustained improvements in well-being were observed after participation in the health program. These results suggest that participation in workplace programs, such as the one evaluated here, also has the potential to improve well-being.

**Electronic supplementary material:**

The online version of this article (doi:10.1186/1471-2458-14-953) contains supplementary material, which is available to authorized users.

## Background

The World Health Organization defines health as "a state of complete physical, mental and social well-being and not merely the absence of disease or infirmity" [1]. As physical activity has been reported to be associated with antidepressant effects [2–4], improved mood [3, 5], biological resilience to psychosocial stressors [2] and employee psychosocial health [4], an increase in physical activity is likely to have a beneficial effect on mental health. Two theories explaining the underlying mechanisms for the relationship between physical activity and psychosocial health have been discussed. The mechanical theory describes changes in the body that affect stress, for example exercise increases the synthesis of brain-derived neurotropic factor which is associated with stress when levels are low [6, 7] and exercise increases body temperature and blood circulation which impacts the hypothalamic-pituitary-adrenal axis and reactivity to stress [3]. The psychological theory relates to self-efficacy and cognitive dissonance. As described by Ikenouchi-Sugita et. al [8], physical activity can relieve feelings of depression, improve social habits and create regular fitness habits which, in turn, improves mood.

A series of physical activity interventions in a range of settings have found beneficial effects for improving mental health and well-being [9–12]. One key setting for health promotion is the workplace, where physical activity programs have attempted to counter balance the gradually increasing sedentary nature of occupations [13–16]. In this setting, health programs that incorporate a pedometer have become a popular method of increasing physical activity as they are low-cost, small, light, portable and easy to-use [16]. These pedometer-based health programs generally involve wearing a pedometer and recording each day’s number of steps (step-count) [17, 18]. As pedometer workplace health programs are increasingly being instigated, it is important to evaluate whether these relatively simple health programs offer an avenue for improving population health. To date, variations of pedometer workplace health programs have reported immediate improvements in physical activity, anthropometric outcomes such as waist circumference and blood pressure, and biomedical outcomes such as total cholesterol [13, 19–39]. It is also of interest whether pedometer-based workplace health programs improve health and well-being more broadly.

Within the context of a pedometer-based work-place health programs, several program characteristics could potentially improve well-being directly. Firstly, as described above, an increase in physical activity is likely to have a direct beneficial effect on mental health through mechanical theory. Secondly, through psychological theory participating in physical activity the participant may increase awareness of fitness capabilities and may increase self-esteem and willingness to participate in other social activities [40]. Thirdly, the pedometer itself provides specific feedback, promoting self-efficacy through Social Cognitive Theory [16, 39, 41–47] via progressive individual goal-setting which allows the participant to be flexible in the amount and the scheduling of their physical activity and provide increased control upon one’s health [16]. Fourthly, if the program has a team component, it creates a social environment for the participant and collegiate camaraderie [8, 16, 46]. Thereby, social cohesion is increased through a focus upon team-based participation and the endorsement of the program by leaders, through both financial and time support, can reinforce participation in such programs. As both social cohesion and the environment are associated with mental health [48], such programs addressing these characteristics may improve well-being directly. Finally, if the program has a competitive component, it may provide increased self-esteem if the participant does well.

Four pedometer-based workplace health program evaluations have encapsulated some of the broader aspects of health [8, 13, 49, 50]. These evaluations have reported differing findings; a decrease in depressive symptoms in previous non-exercisers after a four-week program [8]; no change in Health Related Quality of Life (HRQoL) scales immediately after a nine-week program [13]; an immediate benefit in the mental component of HRQoL after a four-month program [49]; and an immediate benefit by reduction in the number of self-reported physically and mentally unhealthy days after a three-month program, which was sustained fourteen-weeks later [50]. These pedometer workplace health programs have utilised health surveys that either evaluate self-reported depressive symptoms or the impact of positive psychosocial health upon health. It is important to assess general well-being as health promotion programs may have multiple benefits across mental health and well-being and physical health outcomes, not just those related to physical health. General well-being has not previously been evaluated in a pedometer workplace health program.

This paper aimed to evaluate whether participation in a four-month, pedometer-based, workplace health program known to improve biomedical risk factors was also associated with an improvement in well-being. The secondary aim was to identify participant characteristics associated with immediate and long-term improvements in well-being. Potential benefits were assessed immediately after the program (at four-months) and in the longer-term (eight-months after program completion).

## Methods

This paper was undertaken as part of a larger study, titled the Global Corporate Challenge® (GCC®) Evaluation study [17, 49, 51–55].

### Description of the program

The GCC® is a corporate organisation that undertakes a world-wide, annual, four-month (May to September), team-based, visible step-count pedometer, workplace health program. The target is to achieve 10,000 steps per day, which is based upon the World Health Organization’s recommendation [56]. Teams of seven employees enter their step-counts to virtually walk around a world map. Weekly encouragement emails are sent in the form of a newsletter which, in 2008, included the participant’s personal best daily step count, a weekly health tip from a nutritionist, stories from participants, a "Dear GCC" answer to a participant’s question, housekeeping notices and prizes awarded from sponsors. A website was used for logging daily steps, access to additional health information such as the number of steps required to burn off a hamburger, communication among participants and comparing team progress.

### GCC® evaluation recruitment

In 2008, 259 of the workplaces participating in the GCC® had an office located in Melbourne and several were approached by GCC® employees regarding their interest to be study sites for an evaluation. Preference for approaching workplaces was based on early conscription to the GCC® 2008 event, a large number of employees, a variety of sedentary occupations, and the availability of a designated GCC® coordinator. Ten evaluation sites consented, providing access to 4,138 people enrolled in the GCC®. Participating workplaces sent internal emails to inform employees of the evaluation and after interest was registered, Monash University staff sent an email with an explanatory statement and consent form. Seven hundred and sixty two voluntary consenting participants, aged 18 years and above and were recruited and assessed in April/May 2008 [52]. GCC® Evaluation study participants were similar to the other employees enrolled in the GCC® 2008 at the participating workplaces in terms of age and sex, but were more likely to comply with the step goal [51]. At four-months 79% returned [17], and at eight-months after program completion 76% of the original sample returned [51].

### Data collection

Data was collected at baseline, four-months and eight-months after the completion of the program. An Internet self-report questionnaire incorporated demographic information [57–59] (including age, sex, education, household status, marital status and occupation), motivation and support for participation [57] (including the level of support from workplace) and behavioural measures [57, 59, 60] (including tobacco use, alcohol consumption, eating behaviour, physical activity, sedentary behaviour and physical functioning). Measurements were collected by trained staff in the morning at the employee’s workplaces including blood pressure (using Omron IA1B Automatic blood pressure intellisense machines), height (using a stadiometer portable height scale code PE087 and step ladder), weight (using Salter electronic bathroom scales model 913 WH3R 3007 during baseline and four-month data collection and Seca digital scales model Robusta 813 during twelve-month data collection) and waist and hip measurements (using a Figure Finder Tape Measure Novel Products Inc 2005 code PE024 and a mirror).

### Well-being

Well-being was measured using the validated WHO-Five Well-being Index (WHO-5) [61–65], a self-administered five-item scale that is designed to assess subjective well-being. The items cover positive mood (good spirits, relaxation), vitality (being active and waking up fresh and rested), and general interests (being interested in things) [61, 65]. Each of the five items is rated on a 6-point Likert scale and converted proportionally to a score that ranges from 0 (worst thinkable well-being) to 100 (best thinkable well-being) [61, 65]. A score below 52 indicates poor well-being and is an indication for testing for depression under ICD-10 [61]. A 10% difference in the score indicates a clinically significant change [61].

### Variable selection

To compare differences between those with poor versus positive well-being at baseline, baseline measures and average daily step-count throughout the four-month program were assessed. Several variables were dichotomised as meeting commonly accepted clinical guidelines: physical activity (at least 150 minutes of moderate intensity activity per week) [66–78], fruit (two serves a day) [66, 68, 69] and vegetable consumption (four serves a day) [66, 68, 69]. Participants were categorised as meeting tobacco guidelines if they did not report currently smoking or chewing tobacco.

Baseline demographic variables, as well as, the process variable of average daily step-count throughout the four-month program (indicating compliance with the program) were assessed as potential ‘predictors’ of well-being change in response to the program. Psychosocial measures were not considered ‘predictors’ of well-being change due to the potential for introducing bias [70].

### Analysis

Unless otherwise indicated, continuous data is summarised as mean ± standard deviation (SD). Analyses were performed using Stata version 11 [71]. Robust standard errors, clustered by workplace, were used in all statistical analyses. A p-value <0.05 was used to determine statistical significance. Pregnant participants during the study timeframe were excluded from all analyses (n = 28).

#### Retention

Participants who did and did not return at four-months or eight-months after program completion were compared according to baseline variables, four-month change variables and average daily step-count data, where relevant.

#### Well-being change

Change in well-being after participation in the health program was assessed by analysing the difference between baseline and four-month, and the difference between baseline and eight-month after program completion measurements using linear regression. Analyses of change were also stratified by baseline well-being status. The potential magnitude of the regression to the mean effect on the observed change in well-being was estimated using an established method [72]. Positive well-being was assessed for temporal change through conditional logistic regression with time-point as an explanatory variable, where change from poor to positive well-being was represented by a 0, 1 pair of binary values, and positive to poor was represented by a 1, 0 pair [17].

The primary analysis assessing change in well-being associated with participation in the health program included participants who completed the WHO-5 at the three data collection rounds. A secondary (sensitivity) analysis was undertaken using participants with complete data on the relevant two rounds for each analysis of temporal change.

#### Variable selection

Demographic and the process measurement of average daily step count were assessed as potential predictors of immediate and long-term well-being change. Potential predictors were assessed by univariable and multivariable regression analyses with well-being change as the outcome variable.

### Ethics

The study, project number CF08/0271-2008000125, was approved by Monash University Human Research Ethics through the standing committee on ethics in research-involving humans.

## Results

### Retention

Of the 734 eligible participants, 93% (n = 685) completed the WHO-5 at baseline, 64% (n = 468) completed the WHO-5 at baseline and four-month and 55% (n = 407) completed the WHO-5 at all three time-points.

Those who completed the WHO-5 at all three time-points were more likely to be older (mean ± SD: 42 ± 10 years versus 39 ± 11 years, p = 0.002), have a professional position (68% versus 54%, p = 0.001), participate in the GCC® due to appearance reasons (61% versus 54%, p < 0.001), not smoke tobacco (92% versus 86%, p = 0.004), meet baseline physical activity guidelines (42% versus 34%, p = 0.03) and undertake more steps during the health program (11,854 ± 3,777 mean steps per day versus 11,111 ± 3,636, p = 0.02).

### Distribution of well-being

At baseline the distribution of well-being was left skewed, with the majority (75%) being in the positive well-being category. Participants who were older, participating in the GCC® for health reasons, met fruit and physical activity guidelines, had a higher mental quality of life and had undertaken at least 10,000 steps per day on average during the program were more likely to be in the positive well-being category at baseline, Table 1.

**Table 1:** **Comparison of those with poor and positive well-being at baseline** ^**a**^

	Poor well-being (Mean ± SD or Percentage)	Positive well-being (Mean ± SD or Percentage)	P-value ^b^
n	103	304	-
**DEMOGRAPHICS**			
Age (year)	39 ± 9	43 ± 10	**0.004**
Male^c^	42.7	44.1	0.7
Completion of tertiary education^c^	84.5	79.3	0.2
Marital status			
Married or de facto	68.0	71.1	0.5
Widowed, separated or divorced	10.7	10.2	
Never married	21.4	18.8	
**BASELINE MEASURES**			
Prior GCC® Participation^c^	19.4	24.3	0.3
**Motivation for participation**			
Health^c^	78.6	64.5	**0.003**
To look my best^c^	68.0	59.2	0.1
Fitness^c^	73.8	64.8	0.1
Colleagues^c^	56.3	59.5	0.6
Friends or family^c^	1.9	2.3	0.8
**Behavioural measures**			
Fruit Intake^c^ (meeting guidelines)	22.3	37.2	**<0.001**
Vegetable Intake^c^ (meeting guidelines)	15.5	16.8	0.7
Alcohol^c^ (meeting guidelines)	40.8	42.1	0.8
Non smoker^c^	92.2	91.8	0.9
Physical activity^c^ (meeting guidelines)	28.2	46.7	**<0.001**
Sitting time (hrs per day)			
Weekday	8.7 ± 4.0	8.2 ± 3.5	0.2
Weekend	5.6 ± 2.9	5.3 ± 2.9	0.2
**Psychosocial measures**			
Well-being	32.9 ± 13.1	69.3 ± 9.8	**<0.001**
Well-being^c^ (positive category)	0.0	100.0	**-**
Health related quality of life (SF-12)			
Mental health component	38.6 ± 11.0	53.0 ± 6.1	**<0.001**
Physical health component	50.5 ± 8.7	51.2 ± 6.9	0.5
**Anthropometric measures**			
Systolic blood pressure (mmHg)	118.1 ± 12.9	119.6 ± 14.4	0.4
Diastolic blood pressure (mmHg)	79.6 ± 9.2	80.2 ± 10.3	0.6
Heart rate (beats per minute)	68.6 ± 8.5	68.4 ± 10.6	0.8
Weight (kg)	80.8 ± 17.4	76.7 ± 15.2	0.2
Body mass index (kg/m^2^)	27.5 ± 5.0	26.7 ± 4.7	0.3
Waist circumference	90.6 ± 13.0	88.0 ± 12.2	0.2
**STEP DATA**			
Steps average (per day)	11,223 ± 3,515	12,066 ± 3,844	0.06
Meeting 10,000 steps on average^c^ (per day)	63.7	71.7	**0.04**

^a^Restricted to participants who attended baseline, four-month and twelve-month data collection.

^b^Bold highlights statistically significant results.

^c^The reference group for this binary variable is ‘no’. The reference group data is not shown.

### Immediate and long-term well-being change

Immediately after the health program, in the 407 participants with complete data at all three time-points, an average improvement of 3.5 units compared to baseline (p < 0.001) was reported and this was sustained eight-months later (3.4 units, p < 0.001), Table 2. The proportion of participants with positive well-being increased immediately after the program (by 6.2%, p < 0.001), however in the long-term the magnitude of improvement was smaller and not statistically significant (by 2.5%, p = 0.1).

**Table 2:** **Immediate and long-term change in well-being associated with participation in a physical activity workplace program**

		Baseline	Four-month	Twelve-month	Baseline to four-months			Baseline to eight-months post-program		
					Mean change	(95% CI)	P-value	Mean change	(95% CI)	P-value ^a^
Mean (SD)	407	60.1 (19.1)	63.6 (18.6)	63.4 (18.8)	3.5	(2.2, 4.9)	**<0.001**	3.4	(2.0, 4.8)	**<0.001**
Positive well-being (%)	407	74.7	80.8	77.2	6.2	OR: 1.96	**<0.001**	2.5	OR: 1.24	0.1
						(1.48, 2.60)			(0.95, 1.63)	
***Positive baseline well-being***										
Mean (SD)	304	69.3 (9.8)	69.0 (14.6)	68.4 (15.7)	-0.3	(-1.4, 0.8)	0.6	-0.9	(-2.1, 0.2)	0.1
Positive well-being (%)	304	100	91.4	86.5	-8.6	(-11.5, -5.6)	**<0.001**	-13.5	(-19.4, -75.7)	**0.001**
***Poor baseline well-being***										
Mean (SD)	103	32.9 (13.1)	47.8 (20.0)	49.0 (19.8)	14.9	(11.7, 18.0)	**<0.001**	16.1	(12.0, 20.2)	**<0.001**
Positive well-being (%)	103	0	49.5	49.5	49.5	(39.2, 59.8)	**<0.001**	49.5	(38.3, 60.7)	**<0.001**

^a^Bold highlights statistically significant results.

### Change in well-being stratified by baseline well-being

In the 407 participants with complete data at all three time-points, no evidence of immediate or long-term change was found for those with positive well-being at baseline (continuous mean change at four-months: -0.3 units, p = 0.6; eight-months after program completion: -0.9 units, p = 0.1). Significant immediate and long-term improvement was observed for those with poor well-being at baseline (continuous mean change at four-months: 14.9 units, p < 0.001; eight-months after program completion: 16.1 units, p < 0.001). Consequently half of those with poor well-being at baseline moved into the positive well-being category at four-months (49.5%, p < 0.001) and this was sustained at eight-months after program completion (49.5%, p < 0.001). The expected effect of regression to the mean in the group with positive well-being at baseline was that change would be in a negative direction (mean expected change four-months: -3.7 units, eight-months after program completion: -4.7 units), a greater decrease than observed (as described above), Additional file 1. Within the poor well-being group at baseline, regression to the mean anticipated positive changes (mean expected change four-months: 7.4 units, eight-months after program completion: 9.4 units) but the observed change was of a greater magnitude than anticipated (as described above), Additional file 1.

### Sensitivity analysis

When complete case analysis (four-month n = 468, eight-months after program completion n = 496) was undertaken, the magnitude and statistical significance of the changes were similar to the primary analysis; with the attaining of statistical significance for sustained improvement in the proportion with positive well-being (2.5% from baseline to eight-months after program completion, p = 0.02), Additional file 2.

### Predictors of well-being improvement

At four-months, baseline characteristics associated with immediate improvement in well-being were tertiary education and greater number of steps during the program, Table 3. In those with poor well-being at baseline, the characteristics associated with being in the positive well-being category at four-months were older age at baseline and undertaking more steps during the health program, Table 4.

**Table 3:** **Linear regression analyses assessing potential baseline and step-count predictors of four-month well-being change**

Predictor variable	n	Crude well-being change (units)	Univariable		Multivariable model	
			Well-being change (units)	P-value ^a^	Well-being change (units)	P-value ^a^
DEMOGRAPHICS						
Age (year)	407	-	-0.30	0.4	-0.30	0.4
Sex						
Female	229	3.46	Reference			
Male	178	3.66	0.20	0.9	0.23	0.9
Tertiary Education						
Not completed	79	1.11	Reference			
Completed	328	4.13	3.02	**0.03**	3.03	**0.03**
Marital status						
Married/de facto	286	3.13	Reference			
Widowed, separated or divorced	42	8.48	5.34	0.2	5.68	0.1
Never married	79	2.43	-0.70	0.7	-1.09	0.7
PROCESS MEASURES						
Step average per day (per 1,000 steps)	406	-	0.31	**0.002**	0.28	**0.004**

^a^Bold highlights statistically significant results.

**Table 4:** **Linear regression analyses assessing potential predictors of improving from ‘poor’ to ‘positive’ well-being at four-months**

Predictor variable	n	% positive well-being at four-months	Univariable		Multivariable model	
			OR	P-value ^a^	OR	P-value ^a^
DEMOGRAPHICS						
Age (year)	103	-	1.39	**0.008**	1.40	**0.01**
Sex						
Female	59	50.85	Reference			
Male	44	47.73	0.88	0.5	0.85	0.5
Tertiary Education						
Not completed	16	43.75	Reference			
Completed	87	50.57	1.32	0.6	1.43	0.5
Marital status						
Married/de facto	70	51.43	Reference			
Widowed, separated or divorced	11	72.73	2.52	0.2	2.76	0.2
Never married	22	31.82	0.44	0.1	0.56	0.4
PROCESS MEASURE						
Step average per day (per 1,000 steps)	103	-	1.24	**<0.001**	1.22	**<0.001**

^a^Bold highlights statistically significant results.

At eight-months after program completion, baseline characteristics associated with long-term improvement in well-being were younger age and being widowed, separated or divorced, Additional file 3. In those with poor well-being at baseline, the characteristic associated with improvement to the positive well-being category at eight-months after program completion was being widowed, separated or divorced at baseline, Additional file 4.

## Discussion

In this study of 407 adults employed in primarily sedentary occupations and voluntarily enrolled in a physical activity program, 75% of participants were achieving positive WHO-5 well-being scores at baseline. On average, well-being improved immediately after the health program and was sustained eight-months later. A substantial benefit was seen in those with poor well-being at baseline, with around 50% of this group having positive well-being at both four and eight-months after program completion. Achieving a greater number of steps during the program was associated with immediate well-being improvement.

The overall immediate and long-term changes in well-being for the total enrolled population were not clinically significant [61]. This is likely to be due to the high rates of positive well-being observed at baseline (75%). Our mean baseline score of 60 is in the upper range of general population mean scores from various European countries (52 to 68) [73–76] and a group of undergraduate Australian students (mean = 52) [77]. When results were stratified by baseline well-being category, a clinically relevant immediate and long-term benefit was observed for those categorised as having poor well-being at baseline. In a previous analysis assessing potential predictors of immediate improvements in waist circumference in this program, we found, similarly to the current paper, that greater improvement was associated with being in the high-risk category at baseline [54].

The most prominent characteristic associated with improved well-being in the general study population and those with poor baseline well-being was undertaking more steps during the health program. While a few demographic characteristics were associated with well-being change, these were not consistent across analyses. As previously discussed, the program may be having the greatest benefit in those with the greatest opportunity to improve, those who have slightly healthier habits that may find it easier to make the small changes required for a visible outcome and those who achieved the step goal of the health program [54].

Two reviews of workplace physical activity interventions reported insufficient evidence to assess general well-being as an outcome. Conn et. al [78] observed non-significant positive benefits of workplace physical activity interventions upon quality of life and mood, however warned that findings be interpreted with caution given the limited number of included studies. A more recent review by Chu et. al [79] also identified only a few studies assessing general well-being as an outcome of physical activity workplace interventions, which did not provide evidence of a benefit. A pending Cochrane review relating to workplace physical activity may provide further insight [80].

As previously discussed, four pedometer workplace health program evaluations have encapsulated broader aspects of health [8, 13, 49, 50]. One assessed changes in depressive symptoms [8], while three assessed changes in positive psychosocial factors [13, 49, 50] utilising HRQoL scales. Touger-Decker et. al [50] utilised the Centers for Disease Control Healthy Days Surveillance questionnaire (CDC-PDS) [81], which assesses the number of unhealthy days related to self-rated health, physical health, mental health and activity limitations. Puig-Ribera et. al [13] and Harding & Freak-Poli et. al [49] both utilised the SF-12 [82] which assesses affective influences such as physical and subjective emotional feelings. The WHO-5 [61] utilised in our paper assesses affective influences such as subjective emotional feelings and cognitive influences such as satisfaction with the present. The key difference between the WHO-5 [61] is that it assesses general well-being while the SF-12 [82] and CDC-PDS [81] assesses psychological factors specifically related to physical health. Hence, this is the first time well-being independent of physical health has been assessed in a pedometer-based workplace health program evaluation.

Our results support findings from these prior evaluations focusing on health related outcomes that participation in such programs is associated with improvement in positive psychosocial health, especially in those with lower psychosocial health at baseline. In addition, a positive relationship between improvement in positive psychosocial health and physical activity level during a program has also been previously observed. Touger-Decker et. al [50] reported that participation in their three-month pedometer workplace health program reduced summative unhealthy days by 2.2 days (a 23% improvement) directly after the program and this was sustained fourteen-weeks later. Puig-Ribera et. al [13] reported no overall change in HRQoL. However, they reported a non-significant relationship between those with the greatest improvements in physical activity also having the greatest improvements in HRQoL directly after the program. In a previous evaluation of this health program, we demonstrated significant improvement in the mental component score (MCS) of HRQoL [49]. Similarly to Puig-Ribera et. al [13] and this current paper, a greater MCS improvement was observed in those who undertook more physical activity during the program [49]. In addition, we have observed here and in our previous paper [49], that a greater improvement in MCS or well-being was observed in those with a lower baseline level of MCS.

The strengths of this evaluation study included the large sample size and the variety of sedentary occupations within the sample [17, 52]. The main potential limitation of this study is the lack of a control group [17]. Consequently, it is not possible to definitively conclude that improvements observed in this study were attributable to participation in the program [17, 83]. However there is no priori reason to expect well-being to improve over time without an intervention, and by using multiple workplaces, the potential of additional well-being-promoting influences would have been reduced [49]. Additionally, we tested the regression to the mean effect and determined that the change within the positive baseline well-being group was similar, if not better than, the change predicted by the regression to the mean effect. The poor baseline well-being group improved beyond the expected regression to the mean effect. Hence, immediate and long-term improvement in well-being within the poor baseline well-being group is likely to be associated with participation in the health program. Another potential limitation was the assessment of well-being as a secondary outcome of interest in the GCC® Evaluation study which limits the extensiveness of our analysis to the available data, especially given the low prevalence of poor well-being that could be improved via participation in the program. We suggest that future research ensure a sufficient number of participants categorised as having poor well-being to better assess the relationship between well-being and program participation. An additional potential limitation is the lack of assessment of team, program and workplace characteristics, as well as productivity outcomes such as absenteeism, which are not available to assess as predictors in this analysis [54] and these could be explored further in future studies. An important next step is to identify the pathway to the improved mental health, by assessing the influence of specific program characteristics. Another potential limitation is the possible selection bias associated with workplace recruitment, individual recruitment and participant retention. Although enrolees were representative of Australian adults at baseline [52], the healthy volunteer effect may provide healthier employees at the cessation of the evaluation, twelve months from baseline. A healthier, more motivated cohort would be more likely to comply with the health program (overestimating the health benefits) [17] but a greater proportion of a healthier cohort would already be meeting health guidelines at baseline (underestimating the general health benefits of participation due to ceiling effects) [17]. However, selection bias is unlikely to substantially affect the interrelationships between predictors and well-being change. Finally, not obtaining pre-intervention step count is a limitation of this evaluation. At the initiation of the study it was thought that providing a pedometer prior to the initiation of a health program could be confusing to participants. As discussed in this paper, pedometer programs are generally based on Social Cognitive Theory, where self-efficacy is the main driver to positive physical activity and health behaviour change. Hence, provision of a pedometer prior to the initiation of the health program could inadvertently encourage participants to start monitoring their steps and increasing their physical activity. Hence, we felt that obtaining pre-intervention step count may have introduced bias.

## Conclusions

This study is the first to assess the WHO-5 Well-being Index in a working adult population and is the first to assess general well-being, rather than psychological factors specifically related to physical health, in a pedometer-based workplace health program evaluation. At baseline we observed that three quarters of employees were achieving positive well-being scores at baseline. Clinically relevant benefits associated with participation in the health program were observed in the other quarter that had poor well-being at baseline. In addition to anthropometric and biomedical benefits that have been previously observed, psychosocial benefits also appear to be associated with participation in this four-month, pedometer-based, physical activity, workplace health program. These results suggest that a health program such as this which incorporates both physical activity and team involvement has the potential to improve well-being, especially in those who have poor well-being and actively participate in the health program.

## Electronic supplementary material

Additional file 1: **Effects of regression to the mean on changes in the WHO-Five Well-being Index by baseline well-being sub-groups.** (DOC 74 KB)

Additional file 2: **Sensitivity analysis of immediate and long-term change in well-being: complete case data.** (DOC 49 KB)

Additional file 3: **Linear regression analyses assessing potential predictors of improving from ‘poor’ to ‘positive’ well-being at four-months.** (DOC 42 KB)

Additional file 4: **Linear regression analyses assessing potential predictors of improving from ‘poor’ to ‘positive’ well-being at twelve-months.** (DOC 43 KB)
