# Supplementary material for: Change in well-being amongst participants in a four-month pedometer-based workplace health program
Source: BMC Public Health. 2014 Sep 15;14:953. doi: 10.1186/1471-2458-14-953 (PMC4180736; doi:10.1186/1471-2458-14-953)

# Additional file 1: Effects of regression to the mean on changes in the WHO-Five Well-being Index by baseline well-being sub-groups

#
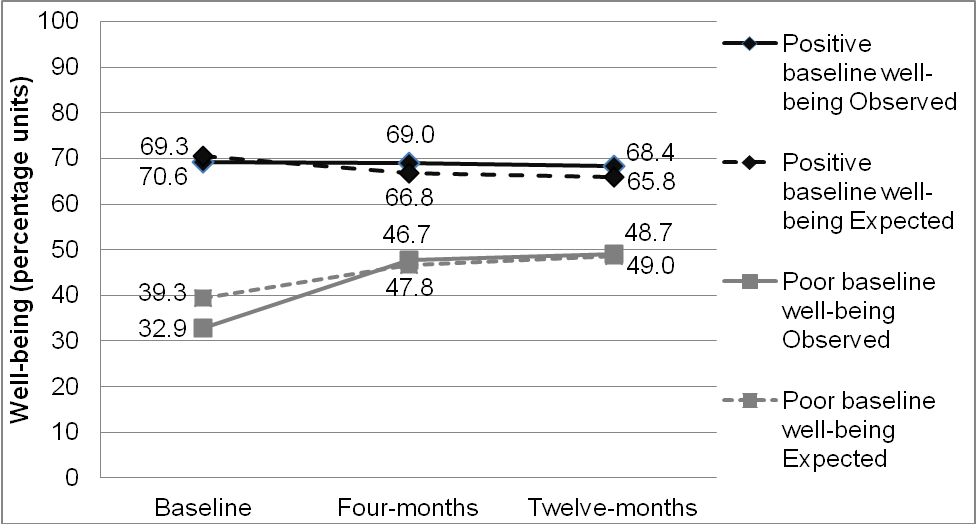

Supplement: Supplementary file 1 — Additional file 1: Effects of regression to the mean on changes in the WHO-Five Well-being Index by baseline well-being sub-groups. (DOC 74 KB) [file 12889_2014_7085_MOESM1_ESM.doc]
