# Supplementary material for: Change in well-being amongst participants in a four-month pedometer-based workplace health program
Source: BMC Public Health. 2014 Sep 15;14:953. doi: 10.1186/1471-2458-14-953 (PMC4180736; doi:10.1186/1471-2458-14-953)
Supplement: Supplementary file 2 — Additional file 2: Sensitivity analysis of immediate and long-term change in well-being: complete case data. (DOC 49 KB) [file 12889_2014_7085_MOESM2_ESM.doc]

# Additional file 2: Sensitivity analysis of immediate and long-term change in well-being: Complete case data

A. Baseline to four-month

|  |  |  |  | **Baseline to four-months** | | |
| --- | --- | --- | --- | --- | --- | --- |
|  | n | **Baseline** | **Four-month** | **Mean Change** | **Difference^a^** (95% CI) | **P-value** |
| Mean (SD) | 468 | 60.0 (19.4) | 63.7 (18.7) | 3.7 | (1.9, 5.5) | 0.001 |
| Positive well-being (%) | 468 | 74.7 | 80.8 | 6.2 | OR: 2.32 (1.70, 3.17) | <0.001 |
| ***Positive baseline well-being*** | | |  |  |  |  |
| Mean (SD) | 343 | 69.3 (9.8) | 69.0 (14.6) | -0.3 | (-1.4, 0.8) | 0.6 |
| Positive well-being (%) | 343 | 100 | 91.8 | -8.2 | (-10.9, -5.4) | <0.001 |
| ***Poor baseline well-being*** | | |  |  |  |  |
| Mean (SD) | 125 | 33.5 (13.1) | 48.5 (19.7) | 15.0 | (11.9, 18.0) | <0.001 |
| Positive well-being (%) | 125 | 0 | 52.0 | 52.0 | (43.9, 60.0) | <0.001 |

**B. Baseline to eight-months post program**

|  |  | **Baseline** | **Eight-months post program** | **Baseline to eight-months post program** | | |
| --- | --- | --- | --- | --- | --- | --- |
|  | n |  |  | **Mean Change** | **Difference^a^** (95% CI) | **P-value** |
| Mean (SD) | 496 | 60.5 (19.2) | 64.0 (18.8) | 3.5 | ( 2.6, 4.3) | <0.001 |
| Positive well-being (%) | 496 | 74.7 | 77.2 | 2.5 | OR: 1.38 (1.04, 1.84) | 0.03 |
| ***Positive baseline well-being*** | | |  |  |  |  |
| Mean (SD) | 368 | 69.8 (10.2) | 69.0 (15.4) | -0.8 | (-2.0, 0.5) | 0.2 |
| Positive well-being (%) | 368 | 100 | 87.2 | -12.8 | (-17.8, -7.9) | <0.001 |
| ***Poor baseline well-being*** | | |  |  |  |  |
| Mean (SD) | 127 | 33.8 (12.6) | 49.4 (20.0) | 15.6 | (13.3, 17.8) | <0.001 |
| Positive well-being (%) | 127 | 0 | 50.8 | 50.8 | (46.2, 55.4) | <0.001 |
