# Supplementary material for: Change in well-being amongst participants in a four-month pedometer-based workplace health program
Source: BMC Public Health. 2014 Sep 15;14:953. doi: 10.1186/1471-2458-14-953 (PMC4180736; doi:10.1186/1471-2458-14-953)
Supplement: Supplementary file 3 — Additional file 3: Linear regression analyses assessing potential predictors of improving from ‘poor’ to ‘positive’ well-being at four-months. (DOC 42 KB) [file 12889_2014_7085_MOESM3_ESM.doc]

# Additional file 3: Linear regression analyses assessing potential baseline, four-month and step-count predictors of eight-months post program well-being change

| Predictor Variable | n | Crude Wellbeing change (units) | Univariate | | Multivariable Model | |
| --- | --- | --- | --- | --- | --- | --- |
|  |  |  | Wellbeing change (units) | P-value | Wellbeing change (units) | P-value |
| DEMOGRAPHICS |  |  |  |  |  |  |
| Age (per 10 years) | 407 | - | -1.29 | **0.02** | -1.31 | **0.01** |
| Sex |  |  |  |  |  |  |
| Female | 229 | 2.97 | reference | |  | |
| Male | 178 | 3.91 | 0.94 | 0.6 | 1.04 | 0.5 |
| Tertiary Education |  |  |  |  |  |  |
| Not completed | 79 | 1.06 | reference | |  | |
| Completed | 328 | 3.94 | 2.88 | 0.1 | 2.60 | 0.2 |
| Marital Status |  |  |  |  |  |  |
| Married or de facto | 286 | 2.21 | reference | |  | |
| Widowed, separated or divorced | 42 | 6.86 | 4.65 | **0.03** | 5.31 | **0.02** |
| Never married | 79 | 5.77 | 3.56 | 0.2 | 2.98 | 0.3 |
| PROCESS MEASURE |  |  |  |  |  |  |
| Step average per day (per 1,000 steps) | 406 | - | -0.18 | 0.2 | -0.17 | 0.2 |
