# Supplementary material for: Change in well-being amongst participants in a four-month pedometer-based workplace health program
Source: BMC Public Health. 2014 Sep 15;14:953. doi: 10.1186/1471-2458-14-953 (PMC4180736; doi:10.1186/1471-2458-14-953)
Supplement: Supplementary file 4 — Additional file 4: Linear regression analyses assessing potential predictors of improving from ‘poor’ to ‘positive’ well-being at twelve-months. (DOC 43 KB) [file 12889_2014_7085_MOESM4_ESM.doc]

**Additional file 4: Linear regression analyses assessing potential predictors of improving from ‘poor’ to ‘positive’ well-being at eight-months post program**

| Predictor Variable | n | % positive well-being at four-months | Univariate |  | Multivariable Model |  |
| --- | --- | --- | --- | --- | --- | --- |
|  |  |  | OR | P-value | OR | P-value |
| DEMOGRAPHICS |  |  |  |  |  |  |
| Age (per 10 years) | 103 | - | 0.94 | 0.8 | 0.94 | 0.8 |
| Sex |  |  |  |  |  |  |
| Female | 59 | 49.15 | reference |  |  |  |
| Male | 44 | 50.00 | 1.03 | 0.9 | 1.04 | 0.9 |
| Tertiary Education |  |  |  |  |  |  |
| Not completed | 16 | 31.25 | reference |  |  |  |
| Completed | 87 | 52.87 | 2.47 | 0.05 | 2.74 | 0.1 |
| Marital Status |  |  |  |  |  |  |
| Married or de facto | 70 | 45.71 | reference |  |  |  |
| Widowed, separated or divorced | 11 | 81.82 | 5.34 | **0.01** | 6.37 | **0.03** |
| Never married | 22 | 45.45 | 0.99 | 1.0 | 1.08 | 0.9 |
| PROCESS MEASURE |  |  |  |  |  |  |
| Step average per day (per 1,000 steps) | 103 | - | 0.99 | 0.9 | 0.98 | 0.8 |
